# Somatosensory Cortical Electrical Stimulation After Reperfusion Attenuates Ischemia/Reperfusion Injury of Rat Brain

**DOI:** 10.3389/fnagi.2021.741168

**Published:** 2021-11-12

**Authors:** Liang-Chao Wang, Wei-Yen Wei, Pei-Chuan Ho, Pei-Yi Wu, Yuan-Ping Chu, Kuen-Jer Tsai

**Affiliations:** ^1^Division of Neurosurgery, Department of Surgery, National Cheng Kung University Hospital, College of Medicine, National Cheng Kung University, Tainan, Taiwan; ^2^Institute of Clinical Medicine, College of Medicine, National Cheng Kung University, Tainan, Taiwan; ^3^Research Center of Clinical Medicine, National Cheng Kung University Hospital, College of Medicine, National Cheng Kung University, Tainan, Taiwan; ^4^Center of Cell Therapy, National Cheng Kung University Hospital, College of Medicine, National Cheng Kung University, Tainan, Taiwan

**Keywords:** cortical electrical stimulation, neuromodulation, ischemic stroke, BDNF, PI3K – PKB/Akt signaling pathway

## Abstract

**Objective:** Ischemic stroke is an important cause of death and disability worldwide. Early reperfusion by thrombolysis or thrombectomy has improved the outcome of acute ischemic stroke. However, the therapeutic window for reperfusion therapy is narrow, and adjuvant therapy for neuroprotection is demanded. Electrical stimulation (ES) has been reported to be neuroprotective in many neurological diseases. In this study, the neuroprotective effect of early somatosensory cortical ES in the acute stage of ischemia/reperfusion injury was evaluated.

**Methods:** In this study, the rat model of transient middle cerebral artery occlusion was used to explore the neuroprotective effect and underlying mechanisms of direct primary somatosensory (S1) cortex ES with an electric current of 20 Hz, 2 ms biphasic pulse, 100 μA for 30 min, starting at 30 min after reperfusion.

**Results:** These results showed that S1 cortical ES after reperfusion decreased infarction volume and improved functional outcome. The number of activated microglia, astrocytes, and cleaved caspase-3 positive neurons after ischemia/reperfusion injury were reduced, demonstrating that S1 cortical ES alleviates inflammation and apoptosis. Brain-derived neurotrophic factor (BDNF) and phosphoinositide 3-kinase (PI3K)/Akt/mammalian target of rapamycin (mTOR) signaling pathway were upregulated in the penumbra area, suggesting that BDNF/TrkB signals and their downstream PI3K/Akt signaling pathway play roles in ES-related neuroprotection.

**Conclusion:** This study demonstrates that somatosensory cortical ES soon after reperfusion can attenuate ischemia/reperfusion injury and is a promising adjuvant therapy for thrombolytic treatment after acute ischemic stroke. Advanced techniques and devices for high-definition transcranial direct current stimulation still deserve further development in this regard.

## Introduction

Ischemic stroke is an important cause of death and disability worldwide (Katan and Luft, 2018). The development of rapid diagnosis and treatment with newer therapies such as intravenous tissue plasminogen activator and thrombectomy has effectively decreased mortality and disability of ischemic stroke over the past decade. However, only limited patients have the opportunity to receive therapy within a narrow therapeutic window. Failure to reperfuse before substantial irreversible injury occurs and results in permanent functional impairment or even death (Goyal et al., 2016). Adjuvant neuroprotective treatment for reperfusion intervention to protect brain tissue from reperfusion injury remains an important part of treatment for ischemic stroke (Shi et al., 2018).

Neuromodulation by electrical stimulation (ES) has been used in the treatment of many chronic central nervous system (CNS) diseases, such as Parkinson’s disease, epilepsy, and psychological disorders (Dougherty, 2018). In ischemic stroke, neuromodulatory strategies, such as transcranial magnetic stimulation, deep brain stimulation, and direct current cortical stimulation, are used for promoting recovery of function (Bao et al., 2020). ES can modulate neuro-regeneration and neuro-plasticity and thus, promote restoration of neurological function after brain injury (Henrich-Noack et al., 2017). Cortical activities induced by ES have been shown to improve functional recovery after ischemic stroke (Adkins et al., 2006; Koesler et al., 2009).

Neuroprotection by ES during the acute stage of ischemic injury has been reported in several studies. Direct ES of specific nuclei in the brain can attenuate ischemic injury as shown in several animal studies. Stimulation at the cerebellar fastigial nucleus was proposed to induce vasodilation, increase neuronal tolerance to depolarization, or decreased the metabolism of penumbra, and thus can reduce infarction area after middle cerebral artery occlusion (MCAO) (Wang et al., 2014). ES of the dorsal periaqueductal gray matter can induce hypertension and vasodilatation and protect the brain against ischemic injury (Glickstein et al., 2003). ES to the cortical surface overlying the ischemic boundary has antiapoptotic, angiogenic, and anti-inflammatory effects through phosphoinositide 3-kinase (PI3K)/Akt signaling pathway and thus can attenuate ischemic injury (Baba et al., 2009). Several studies also showed a neuroprotective effect of non-invasive transcranial direct current stimulation for ischemic stroke. Transcranial direct current stimulation modifies *N*-methyl-D-aspartate receptor expression and inhibits apoptosis and postischemic inflammation to attenuate ischemic injury (Peruzzotti-Jametti et al., 2013; Notturno et al., 2014). The above studies have demonstrated that ES applied in the acute stage of ischemic stroke is a promising adjuvant therapy to attenuate ischemia/reperfusion injury of the brain. However, optimal stimulation target and protocol remain to be explored. Optimal neural stimulation seeks to reduce the amount of charge needed to achieve the target effect and meanwhile reduces tissue damage caused by electric charge. Square biphasic pulse provides a charge-balanced, rectangular, electric pulse to reduce amplitude and charge per phase while preserving stimulation effectiveness and is an effective pattern of brain ES both in animal and human settings (Deprez et al., 2018; De Jesus et al., 2019). Current intensity, stimulation frequency, and pulse width are parameters related to stimulation effect and damage. Electric stimulation at current (0, 100, and 200 μA) and frequency (0, 2, 10, and 50 Hz) had been reported to be safe for rats (Baba et al., 2009). Short stimulus pulse width reduces charge injection and increases the safety range between therapeutic effects and side effects (Kuncel and Grill, 2004). Optimal stimulus parameters differ by therapeutic target and stimulator.

The neuroprotective potential of early sensory stimulation for the treatment of acute ischemic stroke has also been demonstrated in animal studies (von Bornstadt et al., 2018). Several studies support acute sensory stimulation to the affected limbs or whiskers as a potential therapy for ischemic stroke (Lay et al., 2011, 2013; Frostig et al., 2013). The Frostig group reported that mild sensory stimulation of the whiskers protected Sprague-Dawley rats from ischemic injury after MCAO. The proposed mechanism for neuroprotection by early peripheral sensory stimulation is an improvement of collateral circulation to the sensory cortex at the penumbra of the ischemic core by neuronal activities induced by sensory stimulation. Intact neurovascular unit and efficient cortical anastomoses are proposed to be the decisive factors influencing the neuroprotective effect of sensory stimulation. Inappropriate application of sensory stimulation after ischemic stroke may even be detrimental (von Bornstadt et al., 2018). Therefore, further studies to clarify the pros and cons of sensory stimulation are required.

Application of peripheral somatosensory stimulation in rodent model acute ischemic stroke is often confounded by extensive unintended sensory stimulation during experiments. ES of the primary somatosensory (S1) cortex for hand and tongue representations of S1 using intracranial electrodes showed a linear relationship between stimulation current intensity and intensity of sensation in a human study (Kirin et al., 2019). Direct ES of the S1 cortex is a more reliable method to study the neuroprotective effect by sensory stimulation.

Brain-derived neurotrophic factor (BDNF) is an important trophic factor that protects neuronal functions following ischemia, trauma, and toxic brain injury. BDNF was identified as an important mediator of the neuroprotective response to deep brain stimulation (DBS). ES induces the production of BDNF to promote the survival of neuronal cells. Subthalamic nucleus DBS induces the increase in BDNF to support the survival of the nigrostriatal system and promote the functionality of the basal ganglia-cortical circuitry in the parkinsonian brain (Fischer et al., 2017; Fischer and Sortwell, 2019). In an animal model of spinal cord injury, DBS induces the protein expression of BDNF and improves the hind limb motor function (Wang et al., 2020). Upregulation of PI3K/Akt/mammalian target of rapamycin (mTOR) has been reported to play a pivotal role in neuroprotection using pharmacological treatment (Zhou et al., 2010). Activation of BDNF/TrkB signal triggers the downstream PI3K/Akt/mTOR signaling pathway to regulate autophagy and inhibit translocation of Bax from the cytoplasm to mitochondria to support neuronal survival (Chen et al., 2013). The role of BDNF in cortical ES during the acute stage of ischemic stroke deserved further studies.

This study intends to examine the neuroprotective effect of somatosensory ES applied in the acute stage of ischemic stroke using a rodent animal model of transient ischemic stroke. After 90 min of temporal MCAO, rats received 30 min of ES targeting at S1 cortical area starting at 30 min after reperfusion. Neuroprotective effects were evaluated by examining infarction volume, functional outcome, neuronal survival, and apoptosis on post-MCAO day 3. Underlying molecular mechanisms about neuroprotection by ES after transient ischemic stroke were studied. Microglia are key players of inflammation after CNS injury. The impact of cortical ES on postischemic inflammation by the evaluation of migration, proliferation, and activation of microglia was evaluated by co-staining with the pan-microglial marker, ionized calcium-binding adapter molecule 1 (Iba1), and a marker of phagocytic activation, CD68 (Patel et al., 2013). The influence of S1 cortical ES on the BDNF/TrkB/PI3K/Akt/mTOR signaling pathway and autophagy was also studied. This study demonstrates that S1 cortical ES after revascularization is a promising strategy for the treatment of acute ischemic stroke.

## Materials and Methods

### Experimental Design and Animals

**FIGURE 1:**
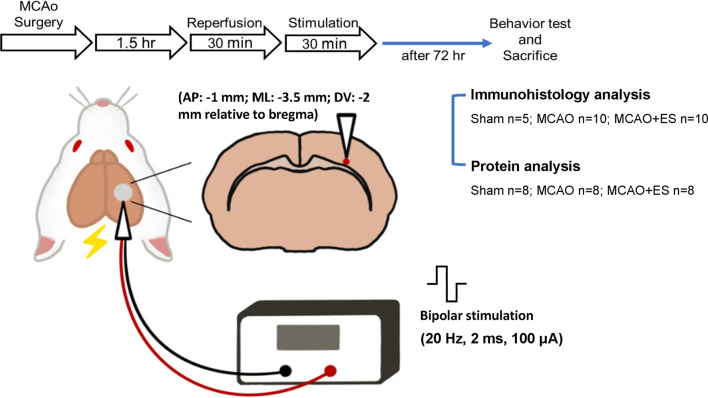
Schematic diagram of the primary somatosensory (S1) cortical electrical stimulation (ES) experimental design. Rats sustained 90 min of transient middle cerebral artery occlusion (tMCAO) or sham operation, and electrodes were implanted to the S1 cortical area. Rats were randomly allocated to three groups and labeled as Sham (sham-operated), MCAO (tMCAO without treatment), and MCAO + ES (tMCAO with ES). A pair of electrodes was stereotactically inserted into the S1 cortex (1 mm posterior to bregma, 3.5 mm lateral to midline, and 2 mm depth) in 30 min after reperfusion. The ES-treated group received 30 min of biphasic direct current ES (frequency, 20 Hz; pulse length, 2 ms; stimulating amplitude, 100 μA; biphasic pulse) after electrode implantation. Functional outcomes were evaluated 3 days after tMCAO, and animals were sacrificed for further studies.

All experiments were conducted under the approval of the Institutional Animal Care and Use Committee (IACUC; Approval #108242) at National Chen Kung University. Adult male Sprague-Dawley rats purchased from the BioLASCO Taiwan Co., Ltd., and weighing 250–300 g were used for all experiments. A total of 60 rats were used in this study: 47 for transient MCAO (tMCAO) and 13 for the sham operation group. In the sham group (13 rats), 5 rats were used for immunohistological analysis and 8 rats were used for protein analysis. The experimental design is illustrated in Figure 1. Rats were randomly allocated to three groups and labeled as Sham (sham-operated), MCAO (tMCAO without treatment), and MCAO + ES (tMCAO with ES). Rats sustained 90 min of tMCAO or sham operation. After reperfusion, a pair of electrodes was stereotactically inserted into the S1 cortex. The ES-treated group received 30 min of ES starting at 30 min after reperfusion. The functional outcome was evaluated at post-tMCAO day 3, and rats were sacrificed for further histology and molecular biology evaluation. In the tMCAO group (47 rats), tMCAO was successfully conducted for 39 rats, whereas 3 of them failed to survive till post-tMCAO day 3. The three mortalities include two in the MCAO group and one in the MCAO + ES group. Thirty-six rats survived till post-tMCAO day 3. Notably, 20 rats (10 for each group) were used for immunohistological analysis and 16 rats (8 for each group) were used for protein analysis.

### Transient Middle Cerebral Artery Occlusion

The MCAO surgery with laser Doppler flowmetry (LDF) monitoring (OxyLab LDF^TM^; Oxford Optronix Ltd., Oxford, United Kingdom) of the cerebral blood flow was conducted as previously described (Boyko et al., 2010; Cao et al., 2017; Wu et al., 2018). Anesthesia was induced with 5% isoflurane mixed with 70% N_2_O and 30% O_2_ in an induction chamber. Surgical-depth anesthesia was maintained with 1–2% isoflurane mixed with 70% N_2_O and 30% O_2_ during surgery. In brief, an area of the skull bone 2 mm posterior and 6 mm lateral to the bregma was decorticated for LDF monitoring. The right common carotid artery and the complex of the internal and external carotid arteries were identified and dissected. The right internal carotid artery (ICA) was further ligated just distal to the bifurcation. The occluders were made of 22 mm long, 4-0 nylon treads. One end of the occluder was heat-treated to expand its diameter from 0.17 to 0.28 mm, thus creating a baseball bat-shaped occluder with a dilated segment of approximately 4 mm in length. The occluder was inserted into the arteriotomy on ICA, advanced until it reached the bifurcation of the anterior and middle cerebral arteries, and left in place for 90 min. A reduction in the cerebral blood flow under 30% of the baseline level was considered a successful occlusion of the middle cerebral artery. After removal of the occluder, a return of the cerebral blood flow to more than 60% of baseline as measured by LDF was considered adequate reperfusion from collateral circulation from the contralateral anterior cerebral artery. For sham surgery, all arteries were exposed for the normal duration of an MCAO operation but the filament was not inserted into the ICA.

### Cortical Electrical Stimulation

Under general anesthesia using 1–2% isoflurane mixed with 70% N_2_O and 30% O_2_, a pair of twisted polyimide-coated stainless-steel bipolar electrodes (E363/3/SPC Invivo1) were stereotaxically positioned to right S1 primary sensory cortex (anteroposterior: −1 mm; mediolateral: −3.5 mm; dorsoventral: −2 mm relative to the bregma) within 30 min after reperfusion. Electrodes were connected to a linear insulation stimulator (WPI, A395), with data acquisition hardware (PowerLab, 8/35) to regulate the output of the stimulator. A 100 μA electric current setting to 20 Hz, 2 ms square biphasic pulse was applied to evaluate the therapeutic effect of short-term intense S1 cortical ES on the acute stage of transient MCAO. Rats received cortical stimulation for 30 min starting at 30 min after reperfusion. In this study, the stimulation intensity used was higher than generally used but 30 min of stimulation is a short one (Bahr Hosseini et al., 2019).

### Neurobehavioral Testing

An 18-point focal scoring system for the modified neurological severity score (mNSS) was used to evaluate neurological outcomes of experimental rats on post-surgery day (PSD) 3 (Chen et al., 2001). Grade scores were assigned to each animal, with functional measures including motor (muscle status and abnormal movement), sensory (visual, tactile, and proprioceptive), reflex, and balance tests (Table 1).

**TABLE 1:** Components of the modified neurological severity score (mNSS) and scoring values.

Motor tests		
Raising rat by tail		Normal = 0; maximum = 3
	Flexion of forelimb	1
	Flexion of hindlimb	1
	Head moved >10° to vertical axis within 30 s	1
Placing rat on floor	Normal walk 0 Inability to walk straight 1 Circling toward paretic side 2 Falls down to paretic side 3	Normal = 0; maximum = 3
Sensory tests		Normal = 0; maximum = 2
	Placing test (visual and tactile test)	1
	Proprioceptive test (deep sensation, pushing paw against table edge to stimulate limb muscles)	1
Beam balance tests	Balances with steady posture 0 Grasps side of beam 1 Hugs beam and 1 limb falls down from beam 2 Hugs beam and 2 limbs fall down from beam, or spins on beam (>60 s) 3 Attempts to balance on beam but falls off (>40 s) 4 Attempts to balance on beam but falls off (>20 s) 5 Falls off; no attempt to balance or hang on to beam (<20 s) 6	Normal = 0; maximum = 6
Reflex absence and abnormal movements		Normal = 0; maximum = 4
	Pinna reflex	1
	Corneal reflex	1
	Startle reflex	1
	Seizures, myoclonus, myodystony	1

### Rotarod Testing

Motor coordination was evaluated in MCAO rats using the rotarod test (Wu et al., 2018). During a training session, rats were placed on an accelerating rotarod (model number: LE8505 Rota-Rod, Panlab Harvard apparatus, Spain), and the running speed of the cylinder was increased slowly from 4 to 40 rpm within 5 min. The rats were trained for 3 days before MCAO. All rats received five separate test trials with 5-min intervals between each trial on PSD 3. The average time of five sessions was recorded for the assessment of motor function.

### Cresyl Violet Staining

To prepare the tissue for immunostaining, rats were deeply anesthetized with 5% isoflurane and transcardially perfused with phosphate-buffered saline (PBS) (Marquardt et al., 2018; Shomer et al., 2020). The brain tissues were postfixed in 4% paraformaldehyde (PFA) overnight, dehydrated, and paraffinized. The sections were collected from the bregma +1 to −3 mm; the paraffinized tissues were sliced at 8 μm per section. For the cresyl violet staining, slides were placed into heated (50°C) 0.1% cresyl violet acetate for 20 min, and the excess stain was rinsed off in running tap water. Finally, the sections were mounted on coverslips with a mounting medium and dried overnight. The quantification of the infarction area was performed using the HistoQuest analysis software version 4.0 (TissueGnostics, Tarzana, CA, United States). The infarction area ratio with correction for edema was evaluated using the following method: infarction area ratio (%) = (contralateral hemisphere - non-infarcted area in the ipsilateral hemisphere) × 100/contralateral hemisphere (Swanson et al., 1990).

### Immunofluorescence Staining

The detailed protocol for immunofluorescence (IF) staining has been described previously (Wu et al., 2018). Rats were deeply anesthetized with 5% isoflurane and transcardially perfused with PBS. The brain tissues were postfixed in 4% PFA overnight, dehydrated, and paraffinized. The sections were collected from the bregma +1 to −3 mm; the paraffinized tissues were sliced at 8 μm per section. For IF staining, antigen retrieval was performed with 0.01 M citrate acid at 100°C for 10–20 min. Then, the sections were blocked with 5% normal donkey serum (Millipore, Temecula, CA, United States) and 0.5% Triton X-100 (Sigma, United States) in PBS. IF staining was performed to detect with anti-NeuN (1:200; Millipore; MAB377), anti-cleaved-caspase-3 (1:200; Cell Signaling Technology, United States; 9661S), anti-Iba1 (1:200; Wako, Japan; 016-20001), anti-CD68 (1:200; Abcam, United Kingdom; AB31630), and anti-glial fibrillary acidic protein (GFAP) (1:300; Millipore; MAB3402). The Alexa Fluor 488-conjugated anti-rabbit secondary antibody (Invitrogen Life Technologies, United States) and Alexa Fluor 594-conjugated anti-mouse secondary antibody (Invitrogen Life Technologies) were used. Finally, the sections were incubated with DAPI and mounted on coverslips with the mounting medium (Dako, Denmark). Sections were examined by fluorescence microscopy (BX51, Olympus, Japan), and the images were captured using a camera coupled to a desktop computer. The number of positive cells and the total stained area were acquired using TissueFAXS and quantified using TissueQuest software version 4.0 (TissueGnostics, Vienna, Austria) as the intensity of immunoreactivity over the set threshold in a blinded manner. Each rat was randomly counted over six ipsilateral cerebral hemisphere views under 20× objective lens from coronal sections.

### Western Blotting

For protein analysis, MCAO rat tissues were prepared from the penumbra and core of the ipsilateral brain marked by 2,3,5-triphenyltetrazolium chloride (TTC) stain and homogenized in radio-immunoprecipitation assay (RIPA) lysis buffer (50 mM Tris–HCl, pH 8.0, 150 mM NaCl, 1% NP-40, 0.5% sodium deoxycholate, 0.1% sodium dodecyl sulfate (SDS)) containing protease inhibitor cocktail (Roche, Switzerland) and the mixture of phosphatase inhibitor cocktail 2/3 (Sigma Aldrich, United States). Then, the extracts were analyzed by 8–12% SDS PAGE, followed by blot hybridization with the following antibodies: Akt (1:3,000; Cell Signaling Technology; 2920), p-Akt (1:1,000; Cell Signaling Technology; 9271), extracellular signal-regulated kinase (ERK) (1:1,000; Millipore; 06-182), p-ERK (1:1,000; Millipore; 05-797), PI3K (1:1,000; Cell Signaling Technology; 4292), p-PI3K (1:1,000; Cell Signaling Technology; 4228), p62 (1:2,000; Abcam; ab56416), LC3B (1:500; GeneTex, United States; GTX127375), and glyceraldehyde-3-phosphate dehydrogenase (GAPDH) (1:5,000; GeneTex; GTX627408). After primary antibody binding, the blots were incubated with the appropriate secondary antibodies and Western Lightning Plus-ECL (PerkinElmer; Waltham, MA, United States) at room temperature. All data were repeated for at least three independent experiments. For quantitative analysis, relative intensities of the bands were normalized to the internal control protein.

### Enzyme-Linked Immunosorbent Assay

For the detection and quantification of protein, rat brain tissues were homogenized in RIPA lysis buffer, and the total protein content was extracted. Notably, 40 μg of total protein from each sample was used to detect BDNF concentration using the specific BDNF ELISA Assay Kit (DBNT00; R&D Systems Inc., Minneapolis, MN, United States). ELISA assay was performed according to the instructions of the manufacturer. All data were repeated for at least three independent experiments.

### Statistical Analysis

All data are statistically analyzed and graphically represented using GraphPad Prism 8 (GraphPad Software, San Diego, CA, United States), which were normally distributed and are presented as the mean ± SE of mean (SEM). Independent experiments with multiple groups were compared with each other using one-way ANOVA followed by *post hoc* Tukey’s test calculated using Prism. The criteria for statistical significance were ^∗^*p* < 0.05, ^∗∗^*p* < 0.01, ^∗∗∗^*p* < 0.001, and ^****^p < 0.0001.

## Results

### Direct Primary Somatosensory Cortical Electrical Stimulation Attenuates Ischemia/Reperfusion Injury and Promotes Functional Outcome Recovery in the Transient Middle Cerebral Artery Occlusion Animal Model

**FIGURE 2:**
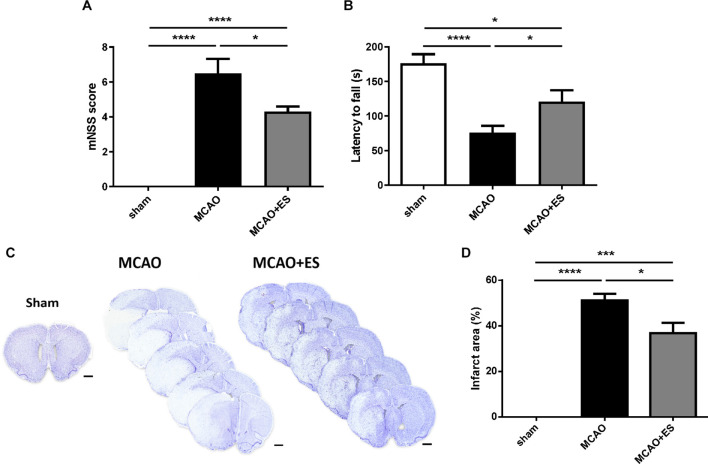
Middle cerebral artery occlusion rats perform better functional outcome recovery and showed reduced infarct area after ES treatment. **(A)** ES attenuates functional deficits as evaluated by mNSS after tMCAO (Sham *n* = 10; MCAO *n* = 10; and MCAO + ES *n* = 10). **(B)** ES improves rotarod performance after tMCAO (Sham *n* = 10; MCAO *n* = 10; and MCAO + ES *n* = 10). **(C)** Representative pictures of crystal violet staining. Scale bar = 1 mm. **(D)** ES significantly reduces infarction volume after tMCAO (Sham *n* = 5; MCAO *n* = 10; and MCAO + ES *n* = 10). *p < 0.05; ***p < 0.001; and ****p < 0.0001.

Infarction volumes were evaluated using cresyl violet staining after tMCAO and 72 h reperfusion. These results demonstrated that S1 cortical ES significantly attenuated infarction volume compared with the control group. Infarction volume decreased from 51.24 ± 2.81% in the MCAO group to 36.79 ± 4.57% in the MCAO + ES group (*p* < 0.05) (Figure 2). Neurological outcomes were assessed by mNSS and the rotarod performance test in tMCAO rats. In line with decreased infarction volume, functional recovery after ischemia/reperfusion injury was also improved in the ES-treated group (Figure 2).

### Primary Somatosensory Cortical Electrical Stimulation Attenuates Neuronal Cell Death in Middle Cerebral Artery Occlusion Rats

**FIGURE 3:**
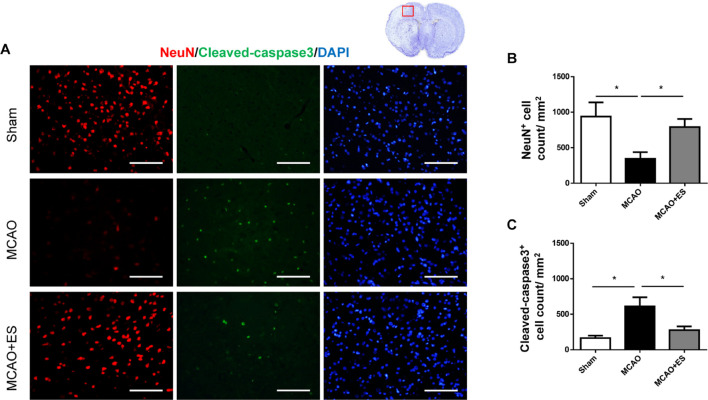
Electrical stimulation treatment inhibits neuronal cell death of the brain in the MCAO animal model. **(A)** Representative pictures of immunofluorescence stains for NeuN (red fluorescence), cleaved caspase-3 (green fluorescence), and DAPI (blue fluorescence). The red blocked box in the top-right panel represents views in the penumbral region. **(B)** Quantitative analysis for NeuN immunoreactive cells. The ES group shows significantly higher neurons survival than the control group. **(C)** Quantitative analysis shows a significantly reduced number of immunoreactive cells for cleaved caspase-3 in the ES group, demonstrating the antiapoptotic effect of ES (Sham *n* = 5; MCAO *n* = 10; and MCAO + ES *n* = 10; scale bar = 50 μm). *p < 0.05.

Whether ES protected neuronal cells from death after tMCAO was further examined. Immunofluorescence staining of NeuN was used to evaluate the survival of neurons in penumbra on day 3 after tMCAO. These results showed the group that received ES after tMCAO had better neurons survival in penumbra as compared with the control group, suggesting that direct S1 cortical ES protects neurons against ischemic damage after tMCAO (Figure 3). Furthermore, cleaved caspase-3 immunofluorescence staining was performed to evaluate apoptosis after tMCAO with or without ES. The elevation of cleaved caspase-3 positive cell count after tMCAO was significantly reduced in the ES group (Figure 3). These data demonstrated that S1 cortical ES had neuroprotective effects and attenuated apoptosis after tMCAO.

### Primary Somatosensory Cortical Electrical Stimulation Inhibits Proliferation and Activation of Microglia and Astrocyte in the Brain of the Middle Cerebral Artery Occlusion Animal Model

**FIGURE 4:**
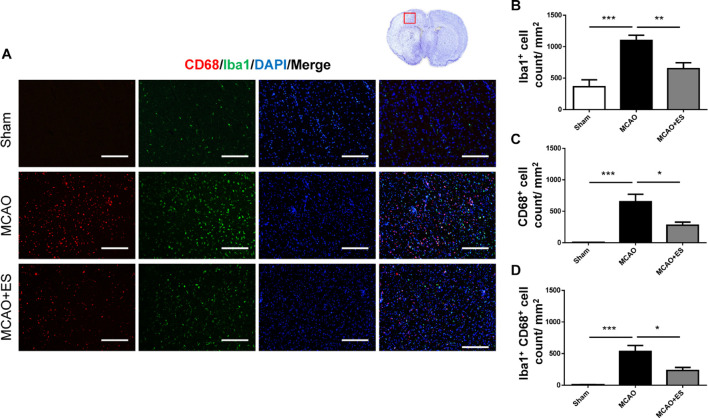
Electrical stimulation treatment affects the activation of microglia in the MCAO animal model. **(A)** Representative pictures of immunofluorescence stains for CD68 (red fluorescence), ionized calcium-binding adapter molecule 1 (Iba1) (green fluorescence), and DAPI (blue fluorescence). The red blocked box in the top-right panel represents views in the penumbral region. **(B–D)** Quantitative analysis of immunoreactive cells. ES treatment significantly reduced proliferation and activation of microglia after tMCAO. Suggesting possible anti-inflammatory effect of ES after tMCAO (Sham *n* = 5; MCAO *n* = 10; and MCAO + ES *n* = 10). Scale bar = 100 μm; *p < 0.05; **p < 0.01; and ***p < 0.001.

**FIGURE 5:**
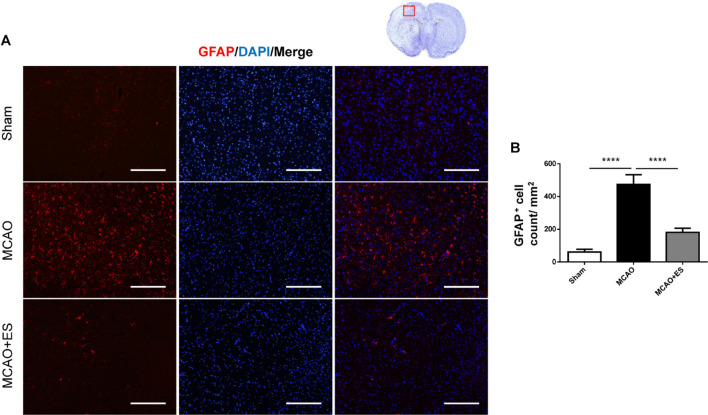
Electrical stimulation treatment reduces the count of astrocytes in the MCAO animal model. Glial fibrillary acidic protein (GFAP) staining demonstrated that proliferation of astrocytes was suppressed by ES. **(A)** Immunofluorescence stains for GFAP (red fluorescence) and DAPI (blue fluorescence). The red blocked box in the top-right panel represents views in the penumbral region. **(B)** Quantitative analysis of immunoreactive cells (Sham *n* = 5; MCAO *n* = 10; and MCAO + ES *n* = 10). Scale bar = 100 μm; ****p < 0.0001.

Activation of microglia/astrocytes plays an important role in the inflammatory response after ischemia/reperfusion insults. To investigate whether neuroinflammation was suppressed by S1 cortical ES, immunofluorescence stains of brain sections were used to evaluate the activation of microglia/astrocytes at 3 days after tMCAO. Activated microglia were identified by co-staining with the pan-microglial marker, Iba1, and a marker of microglial activation, CD68. The number of Iba1/CD68-positive cells in the ischemic penumbra of the cerebral cortex of tMCAO rats was significantly increased than that in the sham group (Figure 4). However, S1 cortical ES significantly reduces microglial activation and the total number of glial cells after tMCAO. Moreover, GFAP staining revealed that the number of astrocytes in the ipsilateral cerebral hemisphere was significantly reduced by ES (Figure 5). Together, S1 cortical ES attenuates proliferation and activation of microglial cells and astrocytes, suggesting the anti-inflammatory effect of ES.

### Primary Somatosensory Cortical Electrical Stimulation Stimulates the Secretion of Brain-Derived Neurotrophic Factor

**FIGURE 6:**
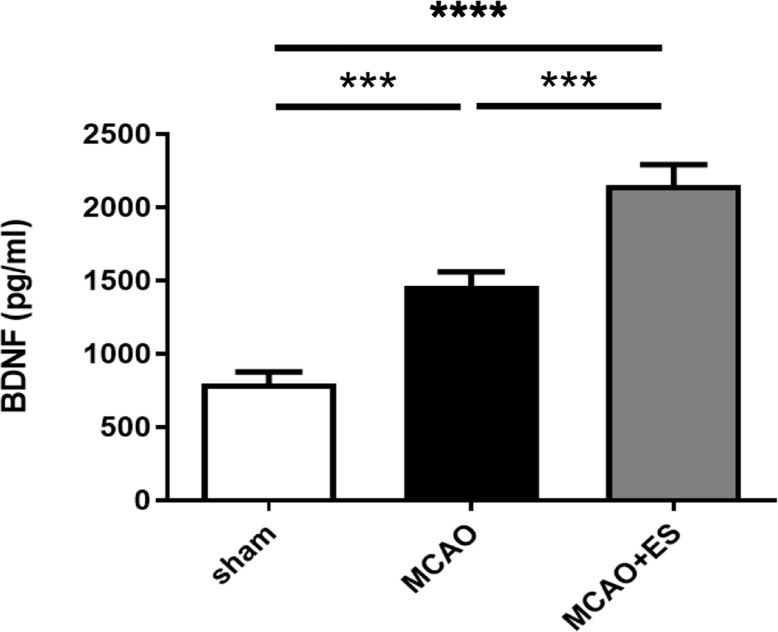
Electrical stimulation treatment enhances the expression of brain-derived neurotrophic factor (BDNF) after tMCAO. Expression of BDNF in penumbra was evaluated by ELISA (Sham *n* = 8; MCAO *n* = 8; and MCAO + ES *n* = 8). ***p ≤ 0.001; ****p ≤ 0.0001.

The BDNF has been reported to play an important role in ES-related neuroprotection. ELISA was used to evaluate the expression of BDNF in penumbra after tMCAO (Figure 6). The data revealed that ES significantly upregulated the expression of BDNF after tMCAO, suggesting that BDNF and its downstream signal pathway play a role in neuroprotection in this ES paradigm.

### Primary Somatosensory Cortical Electrical Stimulation Induces Activation of the PI3K/Akt Pathway in Middle Cerebral Artery Occlusion Rats

**FIGURE 7:**
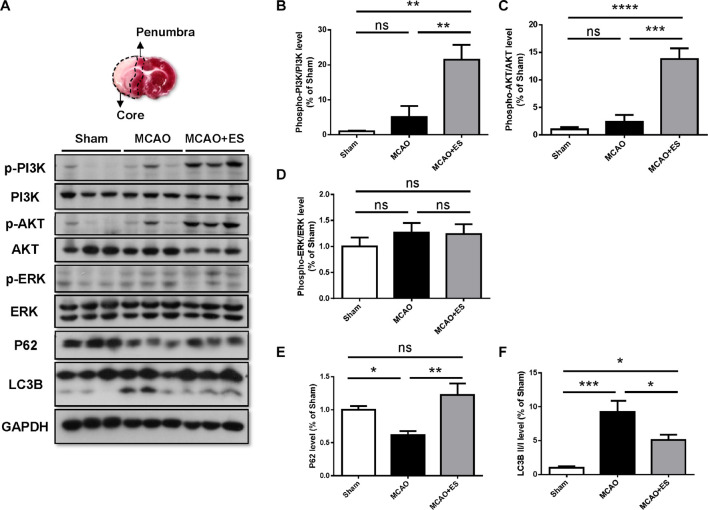
Cortical ES treatment activated the PI3K/Akt pathway and suppressed autophagy to protect the brain from ischemia/reperfusion injury after tMCAO. Western blotting for PI3K, Akt, extracellular signal-regulated kinase (ERK), P62, and LC3B. **(A)** Representative pictures of Western blot. The upper panel represents the 2,3,5-triphenyltetrazolium chloride (TTC)-stained view of demarking core and penumbral region. **(B,C)** Phosphorylation of PI3K and Akt were significantly upregulated in ES groups. **(D)** There was no significant change in the phosphorylation of ERK. **(E,F)** In the ES group, there were increased P62 and reduced LC3B, indicating suppression of autophagy by ES (*n* = 5 for each group). *p < 0.05; **p < 0.01; ***p < 0.001; and ****p < 0.0001.

It has been reported that BDNF modulates autophagy through the PI3K/Akt/mTOR pathway (Nikoletopoulou et al., 2017). Activation of the PI3K/Akt pathway has been reported to promote cell survival and inhibit caspase-3-dependent apoptosis. We hypothesized that S1 ES protects neurons from ischemic/reperfusion injury by the upregulating BDNF/PI3K/Akt signal. To investigate the effect of ES on the PI3K/Akt pathway following an ischemic stroke, Western blotting was performed to examine the expression levels of phosphorylated PI3K, Akt, and ERK in the ischemic brain. Results showed that ES upregulated the levels of phosphorylated PI3K and Akt following tMCAO. However, the change of phosphorylated ERK was not significant (Figure 7). The markers indicating the level of autophagy, p62, and LC3B level were also examined. There was a significant elevation of P62 and reduction of LC3B, suggesting suppression of autophagy by cortical ES. These results demonstrated that ES activated the PI3K/Akt pathway and suppressed autophagy to protect the brain from ischemia/reperfusion injury.

## Discussion

This study demonstrates that 30 min of square biphasic pulse ES at the S1 cortex soon after reperfusion following MCAO attenuates ischemic/reperfusion injury. S1 cortical ES significantly reduced infarction volume, improved functional outcome, and decreased neuronal death and apoptosis. Cortical ES decreased neuronal death and apoptosis. BDNF, PI3K, and Akt were upregulated after ES, demonstrating that BDNF/TrkB and its downstream PI3K/Akt/mTOR signal pathway play a role in neuroprotection by cortical ES.

### Central Nervous System Electrical Stimulation for Neuroprotection in Acute Ischemic Stroke

The neuroprotection effects of CNS ES in acute ischemic stroke have been shown in several preclinical studies. Several strategies for ES have been proposed. In non-invasive transcranial direct current stimulation, a low-voltage electrical current is delivered to the brain *via* scalp electrodes. Despite its easier clinical application, the delivery of electric current is less focused and reliable (Karabanov et al., 2019). Electrical current can also be delivered by electrodes implanted in the brain to provide precise ES. Targets for ES are the fastigial nucleus (Yamamoto et al., 1993), subthalamic vasodilator area (Glickstein et al., 2001), and dorsal periaqueductal gray matter (Glickstein et al., 2003). ES of these deep brain nuclei demands precise targeting and carries the risk of damaging vital structures. Clinical application of these deep brain nucleus stimulation strategies in the acute stage of ischemic stroke is difficult. Cortical ES has the advantage of easier application with less risk. This study demonstrates that cortical ES applied concomitantly with reperfusion could attenuate ischemia/reperfusion injury. The recent development of the high-definition transcranial direct current stimulation technique will make early cortical stimulation a promising treatment for acute ischemic stroke.

### Electrical Stimulation to the Sensory Cortex in the Acute Stage

Sensory stimulation has been proposed as a potential therapy for ischemic stroke. Ron Frostig’s group from the University of California Irvine reported the therapeutic effect of whisker stimulation after permanent MCAO (pMCAO) (Frostig et al., 2013). Mild sensory stimulation of the whisker(s) protected Sprague-Dawley rats from neuronal damage and functional loss after pMCAO (Lay et al., 2011, 2012). It was hypothesized that early sensory stimulation induces cortical activation, which plays a key role in sensory stimulation-related neuroprotection (Lay et al., 2010). After acute vascular occlusion, collateral blood flow through anastomoses between cerebral arteries is important to rescue the tissue from ischemic injury (Cook et al., 2012). Under physiological conditions, increased neuronal activity induces regional vasodilation according to a process of neurovascular coupling that increases the delivery of oxygen and nutrients. In the penumbra area of ischemia where blood supply is decreased but the neurovascular coupling is still relatively preserved, the increased neural activity may attenuate ischemic injury by inducing vasodilatation and blood perfusion. However, in areas where the neurovascular coupling is lost, the increased neural activity is detrimental due to increased energy consumption without corresponding incremental blood flow (Krainik et al., 2005). The timing of stimulation, the vascular anatomy, and especially efficient cortical anastomoses between the cerebral arteries was proposed to be the decisive factors influencing the neuroprotective effect of sensory stimulation (von Bornstadt et al., 2018). In this study, the animal model of tMCAO was used by ligation of ipsilesional ICA, used heat-shaped filament to occlude the origin of middle cerebral artery to minimize collateral blood supply, and then stimulated directly on the cortical S1 area after removal of the occluder for reperfusion. This study design minimized the impact of stimulation time and collateral circulation on the neuroprotective effect. Neurovascular coupling in penumbra is also an important factor for the spread of ischemic injury. Depolarizing events during focal ischemia, such as anoxic depolarization and peri-infarct spreading depolarizations, may result in the vasoconstrictive form of neurovascular coupling and deteriorated blood flow in penumbra (Shin et al., 2006). In the rat model of stroke, cathodal transcranial direct current stimulation in the rat stroke model had been reported to decrease the number of depolarization and attenuate infraction volume (Notturno et al., 2014). Vagus nerve stimulation can also reduce spreading depolarization burden and cortical infarct volume (Lindemann et al., 2020). This study applies ES to the penumbra S1 cortex during the acute stage of tMCAO. Whether S1 cortical ES protects ischemia/reperfusion by influencing postischemic depolarization and neurovascular coupling remained to be explored. The proposed influence of vasodilation induced by ES cannot be excluded in these experiments.

### Neuroprotection Through the Brain-Derived Neurotrophic Factor/TrkB Signal Pathway and Suppression of Autophagy

The BDNF plays an important mediator of the neuroprotective response by DBS. Neuron, astrocyte, and microglia are all possible sources of BDNF. BDNF mRNA is expressed by numerous nuclei in the basal ganglia, and the secretion of BDNF was reported to play an important role in neuroprotection by subthalamic nucleus DBS (Fischer and Sortwell, 2019). The abundance of BDNF transcripts can be found in both glutamatergic and GABAergic neurons from the hippocampus and cortex (Barreda Tomás et al., 2020). However, changes in BDNF expression under pathological conditions are also not fully understood. ES can induce the release of native BDNF, and high-frequency ES (25 and 100 Hz) is more effective at releasing BDNF than low-frequency stimulation (5 and 10 Hz) (Balkowiec and Katz, 2002). This study shows that 30 min of short-term somatosensory cortical ES at a frequency of 20 Hz soon after reperfusion increases the secretion of BDNF. Downstream signal pathways of phosphorylated PI3K and Akt were also upregulated. These results suggested that the BDNF/TrkB signal pathway also plays a role in ES-related neuroprotection during acute ischemia/reperfusion injury.

Autophagy has been proposed as a “double-edged sword” during ischemic injury. Acute and severe ischemia causes excessive autophagy and promotes neuronal damage. Chronic and mild ischemia triggers moderate autophagy to remove damaged proteins and supports neuronal survival (Morselli et al., 2008). Beclin 1, LC3-phosphatidylethanolamine conjugates (LC3-II), and P62 are the three major proteins indicating the level of autophagy. This experiment also showed cortical ES upregulated P62 and suppressed LC3B expression, suggesting that the suppression of autophagy plays a role in cortical ES-related neuroprotection. The detailed mechanisms for the change of BDNF were not studied in these experiments.

### Anti-inflammatory Effects of Cortical Electrical Stimulation

Inflammation and autophagy play an important role in the survival of nerve cells and the recovery of neural tissue after ischemia/reperfusion injuries (Mo et al., 2020). Anti-inflammation was also proposed as a possible mechanism of neuroprotection by cortical ES (Baba et al., 2009). Microglia are key players of inflammation after CNS injury. In this study, the activation of phagocytic microglia after ischemia was suppressed by cortical ES, as presented by the decreased number of Iba1/CD68-positive cells in the ischemic penumbra of the cerebral cortex. The result suggested the anti-inflammatory effect of cortical ES. However, the impact of cortical ES on postischemic neuroinflammation was not studied in detail. Activated microglia and infiltrating macrophage are the main phagocytic cells that remove dead cells. In this study, decreased microglial activation may just be a reflection of decreased neuronal death by neuroprotection after ES.

### Primary Somatosensory Cortical Electrical Stimulation Is a Promising Adjuvant Therapy for Revascularization Treatment

Early revascularization therapy, such as thrombolysis or thrombectomy, is the current treatment of choice for acute ischemic stroke. However, the narrow therapeutic window limits the outcome of these therapies. Adjuvant therapies are required to protect the brain tissue and prolong the therapeutic window. Several preclinical studies demonstrated that CNS ES is a potential acute neuroprotective intervention for ischemic stroke. This study demonstrates the neuroprotective effect of sensory cortical ES applied at the time of reperfusion after occlusion of the middle cerebral artery. These results showed the potential of ES as adjuvant therapy for revascularization treatment after acute ischemic stroke. However, implantation of electrodes is an invasive procedure that may be an obstacle during the clinical application of ES in the acute stage of stroke. The recent development of non-invasive methods for ES, such as high-definition transcranial direct current stimulation, will help the application of early ES during the acute stage of ischemic stroke. The results of this study provide a possible neuroprotective strategy concomitant with revascularization therapies to improve the outcome of acute ischemic stroke.

## Conclusion

This study demonstrates that S1 cortical ES is a promising adjuvant neuroprotective therapy for revascularization treatment during the acute stage of ischemic stroke. Advanced techniques and devices for high-definition transcranial direct current stimulation still deserve further development in this regard.

## Data Availability Statement

The original contributions presented in the study are included in the article/supplementary material, further inquiries can be directed to the corresponding author/s.

## Ethics Statement

The animal study was reviewed and approved by the Institutional Animal Care and Use Committee (IACUC) at National Chen Kung University.

## Author Contributions

K-JT and L-CW: conceptualization, investigation, funding acquisition, project administration, resources, supervision, and writing – review and editing. W-YW, P-CH, and P-YW: data curation. W-YW, K-JT, and L-CW: methodology. W-YW, P-CH, K-JT, and L-CW: validation. L-CW, Y-PC, and W-YW: writing – original draft. All authors contributed to the article and approved the submitted version.

## Conflict of Interest

The authors declare that the research was conducted in the absence of any commercial or financial relationships that could be construed as a potential conflict of interest.

## Publisher’s Note

All claims expressed in this article are solely those of the authors and do not necessarily represent those of their affiliated organizations, or those of the publisher, the editors and the reviewers. Any product that may be evaluated in this article, or claim that may be made by its manufacturer, is not guaranteed or endorsed by the publisher.

## Abbreviations

Akt: protein kinase B
BDNF: brain-derived neurotrophic factor
DBS: deep brain stimulation
ELISA: enzyme-linked immunosorbent assay
ERK: extracellular signal-regulated kinase
ES: electrical stimulation
GAPDH: glyceraldehyde-3-phosphate dehydrogenase
GFAP: glial fibrillary acidic protein
Iba1: ionized calcium-binding adapter molecule 1
ICA: internal carotid artery
LC3-II: LC3-phosphatidylethanolamine conjugates
LDF: laser Doppler flowmetry
mTOR: the mammalian target of rapamycin
mNSS: modified neurological severity score
PBS: phosphate-buffered saline
PFA: paraformaldehyde
PI3K: phosphoinositide 3-kinase
PSD: post-surgery day
RIPA: radio-immunoprecipitation assay
SDS: sodium dodecyl sulfate
SEM: standard error of mean
tMCAO: transient intraluminal occlusion of middle cerebral artery
TTC: 2,3,5-triphenyltetrazolium chloride.
